# Long–Chain Saturated Fatty Acids in Olive Diacylglycerol Stearin Enhances Resistant Starch Content of Candelilla Wax Oleogel Cookies

**DOI:** 10.3390/foods13162589

**Published:** 2024-08-19

**Authors:** Xiaohan Chen, Xiaoxia Chen, Daoming Li, Weifei Wang

**Affiliations:** 1 Department of Food Science and Engineering, School of Food Science and Engineering, South China University of Technology, Guangzhou 510640, China; cxhan1111@163.com; 2 Department of Bioscience and Bioengineering, South China University of Technology, Guangzhou 510640, China; cxxxxxia@163.com; 3 School of Food and Biological Engineering, Shaanxi University of Science and Technology, Xi’an 710021, China; dmli@sust.edu.cn; 4 Sericultural & Argi-Food Research Institute, Guangdong Academy of Agricultural Sciences, Guangzhou 510640, China; 5 Key Laboratory of Functional Foods, Ministry of Agriculture and Rural Affairs, Guangzhou 510640, China; 6 Guangdong Key Laboratory of Agricultural Products Processing, No. 133 Yiheng Street, Dongguanzhuang Road, Tianhe District, Guangzhou 510610, China

**Keywords:** diacylglycerol stearin, candelilla wax, oleogel, starch digestibility, cookie

## Abstract

The purpose of this study was to substitute shortening with olive diacylglycerol oil/candelilla wax (OCW)–olive diacylglycerol stearin (ODS) oleogels and evaluate their impact on starch digestibility in cookies. The in vitro digestibility study confirmed that the OCW/ODS–based cookies exhibited a notable enhancement of 14.6% in slowly digestible starch (SDS) and an increase of 3.14% in resistant starch (RS) values when contrasted with shortening cookies. The XRD pattern indicated that the existence of ODS may improve the formation of complexes between lipids and amylose. The DSC analysis demonstrated that the incorporation of ODS led to a remarkable rise in enthalpy alteration, escalating from 0.90 to 437.70 J/g, suggesting an improved ability to resist gelatinization. The FTIR spectra suggested that the incorporation of ODS might strengthen interactions between the hydrogen bonds and form the short–range ordered structure in OCW/ODS–based cookies. Overall, these results indicated that incorporating OCW/ODS–based oleogels could serve as a feasible substitute for conventional shortening in cookies with decreased starch digestibility.

## 1. Introduction

Cookies are widely consumed globally, with wheat flour (71–84% amylose) and shortening serving as the fundamental constituents [1]. However, the shortening comprises a significant amount of saturated and trans fatty acids, posing potential health risks for humans [2,3]. Consequently, oleogels are considered healthful substitutes for shortenings due to their minimal levels of saturated fatty acids and no *trans* fatty acids [4]. Candelilla wax (CW) is extensively employed as an oleogelator in oleogel formulations [5] and has been acknowledged by the Food and Drug Administration (FDA) as generally accepted as safe (GRAS) [6,7]. However, Barragán-Martínez, et al. [8] recently discovered that substituting shortenings with CW–based oleogels leads to increased digestibility of starch in cookies. The enhanced digestibility of starch is strongly linked to the rise and prevalence of diverse health issues in humans, such as obesity [9] and diabetes [10]. Therefore, the reduction of starch digestibility holds utmost significance for CW–based oleogel cookies.

Olive diacylglycerol stearin (ODS) is obtained through the dry fractionation of olive diacylglycerol oil (ODO), which exhibits elevated levels of long–chain saturated fatty acids [11]. The incorporation of ODS in bakery products may potentially reduce their digestibility by forming amylose–lipid complexes with starch. The molecular dynamics simulation conducted by Feng, Junejo, Zhang, Fu and Huang [12] revealed that amylose tends to form lipid–amylose complexes with distearoyl–glycerol through hydrophobic interactions. Chen, Lan, Li, Wang and Wang [13] also reported that peanut diacylglycerol stearin could form amylose–lipid complexes with starch modified by octenyl succinic anhydride (OSA). These lipid–amylose complexes, which have a compact and ordered structure, result in a decrease in the vulnerability of enzymes to decompose, ultimately resulting in a decline in the digestibility of starch [14]. However, the formation of lipid–amylose complexes may depend on the proportion of long–chain saturated fatty acids. Zhou, Guo, Gladden, Contreras and Kong [15] discovered that starch has a greater inclination to form complexes with octadecanoic acid. Although numerous studies have been carried out regarding the development of lipid–amylose complexes between long–chain saturated fatty acids and amylose, limited investigations have been carried out to explore the influence of long–chain saturated fatty acids in ODS on the formation of lipid–amylose complexes. Moreover, there is also a lack of studies on the correlation between lipid–amylose complexes and the digestibility of cookies made with CW–based oleogel.

The aim of this study was to evaluate the impact of long–chain saturated fatty acids in olive diacylglycerol stearin on the starch digestibility of CW–based cookies. The impact of oleogels on the digestibility of cookies was examined using in vitro digestion analysis. The fundamental mechanism of oleogels’ influence on the digestibility of cookies was investigated using various analytical methods, including X–ray diffraction (XRD), differential scanning calorimetry (DSC) and Fourier transform infrared spectroscopy (FTIR).

## 2. Materials and Methods

### 2.1. Materials

Olive diacylglycerol oil (16.34% saturated long–chain fatty acid, 15.12% TAG, 55.96% 1,3–DAG, 29.52% 1,2–DAG, 0.19% MG, 0.21% FFA) and olive diacylglycerol stearin (26.06% saturated long–chain fatty acid, 12.02% TAG, 68.59% 1,3–DAG, 19.16% 1,2–DAG, 0.14% MG, 0.10% FFA) were acquired by Guangdong Yue–shan Special Nutrition Technology Co., Ltd. (Guangzhou, Guangdong, China), candelilla wax (a natural mixture of hydrocarbon compounds such as free fatty acids and alcohols along with wax esters, ketones and sterols) was acquired by Yuanye Bio–Technology Co., Ltd. (Shanghai, China), shortenings (42.60% saturated long–chain fatty acid) and wheat flour (amylose content ranging from 71% to 84%) were acquired at a nearby supermarket. All the remaining analytically pure chemical reagents were supplied by Aladdin Biochemical Technology Co., Ltd. (Shanghai, China). Pure water was employed in all formulations.

### 2.2. Oleogel Preparation

The preparation of the oleogel was conducted using a method outlined by Barragán-Martínez, et al. [8], with some modifications. Different amounts of CW and ODS were added to olive diacylglycerol oil (5–0%; 5–5%; 5–15%; 5–25%; 5–35% based on ODO) and heated at a constant stirring temperature of 90 ℃ for a duration of 2 h. Afterward, the oleogels were placed in a refrigerator set at a temperature of 4 °C for a duration of 24 h. Prior to analysis, the oleogels were transferred to an environment with a temperature of 20 °C for 1 h. The designated names given to these oleogels were OCW, OCW/ODS–5, OCW/ODS–15, OCW/ODS–25 and OCW/ODS–35 depending on the concentration levels of CW and ODS used in each case.

### 2.3. Cookies Preparation

The cookie was made following the procedure outlined by Barragán-Martínez, et al. [8], with some modifications. The flour weighed 20 g. The formulation comprised baking soda (1% *w*/*w*), milk powder (1.5% *w*/*w*), sugar (45% *w*/*w*), salt (1% *w*/*w*) and water (11% *w*/*w*). After thorough mixing, 40% *w*/*w* of various types of oleogels were separately incorporated into the mixture. Subsequently, the blend was mixed for a duration of 90 s before adding wheat flour and ensuring complete integration. Using a cookie mold, the dough was shaped into discs measuring 50 mm in diameter and 6 mm in thickness. These dough slices were then baked at an oven temperature of 180 °C for a period of 12 min. For comparison, a shortening–based cookie was also prepared using an identical procedure with the substitution of shortening for oleogels. The cookies were categorized into different types based on the specific oleogels used, including shortening cookies, OCW cookies, OCW/ODS–5 cookies, OCW/ODS–15 cookies, OCW/ODS–25 cookies and OCW/ODS–35 cookies.

### 2.4. Fatty Acid Profile

The fatty acid composition and trans fatty acids were analyzed using an Agilent 7890A GC system (Agilent Technologies, Palo Alto, CA, USA) equipped with a capillary CP–Sil 88 column (60 m × 0.25 mm, 0.2 µm film thickness). To prepare the sample for analysis, a previously described method [16] was followed to convert it into fatty acid methyl esters (FAMEs) through methylation. The oil (60 mg) was dissolved in isooctane solution (4 mL), and then methyl esterification was carried out by adding potassium hydroxide–methanol solution (200 µL). Subsequently, the sample underwent GC analysis with a split ratio of 30:1. The detector was operated at a temperature of 280 °C, while the initial temperature was set to 250 °C. The column temperature underwent a gradual increase from 140 °C to 200 °C over a period of 2 min, followed by a slower increase to reach 220 °C in the next 18 min. Finally, it was raised to the final temperature of 230 °C within 10 min. To identify fatty acid methyl esters (FAMEs), their retention times were compared with established standards and quantified using the peak area normalization method [17].

### 2.5. Peroxide Value (PV)

The cookies were placed in polyethylene food–sealed bags and incubated at temperatures of 25 °C, 35 °C and 45 °C for a duration of 35 days. Samples were collected every 7 days, and the PV was determined following the guidelines outlined in GB5009.227–2016 [18]. Triplicate samples were prepared at each storage temperature, with each replicate being analyzed twice concurrently.

### 2.6. Scanning Electron Microscopy (SEM) of Cookies

The scanning electron microscope (LEICA, model LEO440i, London, UK) was utilized to analyze the microstructure of cross–sections from different types of cookies. To enhance the observation quality, a thin layer of gold was sputtered onto 1 mm thick cookie slices for 30 s under an accelerating voltage condition of 3 kV. Following this, images were captured at a magnification level of 100.

### 2.7. The Content of Total Starch

The content of total starch (TS) in cookies was determined using a modified version of the method outlined by Liu, et al. [19]. To prepare the sample, cookies were ground and mixed with three times the amount of water. A wet sample was obtained. The wet sample was subsequently combined with 8 mL of a sodium hydroxide solution (1 mol/L) and agitated for 20 min at a temperature of 25 °C, with a stirring rate of 180 r/min. Finally, 20 mL of a pH 4.7 acetate–sodium acetate buffer with a concentration of 100 mol/mL was added. A 5 mL mixture was placed into a 15 mL centrifuge tube, and then pH 7.4 phosphate buffer solution with a concentration of 50 mol/mL and α–amylase solution with a concentration of 25 mg/mL were added to a volume of 3 mL. The mixture obtained was subjected to agitation in a water bath set at precisely 37 °C for exactly 45 min while rotating at 200 r/min. After the reaction was finished, a 200 μL sample was taken out to measure the glucose content using the GOPOD kit. Ultimately, a conversion coefficient of 0.9 was established for starch to glucose.

### 2.8. In Vitro Digestibility

The method described by Alvarez–Ramirez, et al. [20] was employed with minor adjustments to analyze the starch fractions during digestion. In particular, 1 g of cookies was weighed and then mixed with 10 mL pH 5.2 acetate–sodium acetate buffer with a concentration of 0.25 mol/L. The amylglucosidase was added at a concentration of 1200 mg/mL, along with pepsin at a concentration of 120 mg/mL and porcine pancreatic α–amylase at a concentration of 50 mg/mL. The reaction was then carried out for a duration of 3 h, under agitation at 160 r/min in an air bath shaker. At specific time intervals during the digestion process (0, 20, 60, 90, 120 and 180 min), a sample of enzyme solution measuring 1 mL was extracted for further analysis. To halt the enzyme activity, anhydrous ethanol (85%) in a quantity of 4 mL was introduced and then subjected to centrifugation at a speed of 3500 r/min for 5 min. The resulting liquid above the sediment was then subjected to analysis of the content of glucose using GOPOD kits. Formulas (1)–(4) were employed to calculate the values pertaining to rapidly digestible starch (RDS), slowly digestible starch (SDS) and resistant starch (RS), as well as the rate at which starch hydrolysis occurred during digestion.

(1) $$\mathrm{RDS}\;(\%)=\frac{(G_{20}{-G}_{0})\times 0.9}{TS}\times 100$$

(2) $$\mathrm{SDS}\;(\%)=\frac{(G_{120}-G_{20})\times 0.9}{TS}\times 100$$

(3) RS (%)=100 − RDS (%) − SDS (%)

(4) $$\mathrm{Hydrolysis}\;\mathrm{rate}\;(\%)=\frac{G_{t}\times 0.9}{TS}\times 100$$

where G_0_ indicates the content of glucose; G_20_ and G_120_ denote the glucose contents at 20 min and 120 min during the digestion process, respectively; G_t_ represents the glucose content at any given time of digestion.

### 2.9. X–ray Diffraction (XRD)

The X–ray diffractometer (D8 Advance, Bruker, Germany) was utilized to acquire the XRD patterns of different categories of cookies. The angle of diffraction (2θ) utilized for the cookies ranged from 5 to 50°. Cu–Kα radiation at 40 mA and 40 kV was employed, with a scan rate of 0.02°/s. The scanning rate was modified to attain a velocity of 12°/min when measuring at 2θ. To determine the percentage of crystallinity in complexes formed by lipids and amylose, JADE software 2022 was employed to calculate the proportion of diffraction peak area at approximately 13.3°, 19.8° and 20°.

### 2.10. Differential Scanning Calorimeter (DSC)

The thermal characteristics of the cookies were evaluated using a modified version of the methods outlined by Kaur, et al. [21]. The differential scanning calorimeter (Q–200, Thermal Analyzers Instruments, Washington, DC, USA) was utilized to analyze the heating profiles of cookies. The 8 mg cookies were combined with 16 µL of pure water in the DSC crucible. The crucible allowed us to reach equilibrium over a period of 12 h. Afterward, the sample was performed in the temperature range of 20 °C to 100 °C. The heating rate varied from 20 °C to 100 °C/min. The enthalpy change (∆H_m_), end temperature (T_c_), peak temperature (T_p_) and initial temperature (T_o_) were documented during the process of melting.

### 2.11. Fourier Transform Infrared (FTIR) Spectroscopy

The interaction of the cookie was revealed using a modified version of the method described by [20]. FTIR spectra of different cookie types were obtained using an FTIR spectrophotometer (Vertex 70 V, Bruker, Germany). The spectral data was acquired within the frequency range of 4000–400 cm^−1^. The infrared spectra were processed using Omnic 9.2 software. The half–peak width was set to 30 cm^−1^ and the enhancement factor was 2. The heights at specific wavenumbers of 1047 cm^−1^, 1022 cm^−1^ and 995 cm^−1^ were determined by performing deconvolution integration.

### 2.12. Statistical Analysis

The trials were carried out three times, and then the mean value was computed. SPSS (Version 25) software was used to calculate the statistical analysis, employing one–way analysis of variance (ANOVA) followed by Duncan’s post hoc test (*p* < 0.05). The ORIGIN software 2024b was utilized for data visualization, with distinct letter labels indicating noteworthy differences.

## 3. Results and Discussion

### 3.1. Fatty Acid Composition of Stortening and Oleogels

The fatty acid composition and the total amount of fatty acids in shortening, ODO, ODS and OCW/ODS–based oleogels used in cookies are presented in Table 1. The main components of shortening were palmitic acid (C16: 0) and oleic acid (C18: 1n9c), which accounted for 32.48% and 20.65%, respectively. The content of saturated fatty acid (SFA) in shortening was significantly higher at 69.83% compared to the ODO, ODS and OCW/ODS–based oleogels. The content of *trans* fatty acid (C18: 1n9t) was found to be 3.73%, which poses potential health risks [22]. ODO and ODS mainly consisted of oleic acid (C18: 1n9c), with percentages reaching 69.69% and 63.26%, respectively. A significant increase in the content of palmitic acid (C16: 0) was observed, with a rise from 12.82% (ODO) to 20.24% (ODS), when comparing the fatty acid profiles between ODO and ODS. This resulted in an increase in SFA content in ODS as well [23]. No *trans* fatty acids were detected in both ODO and ODS. Furthermore, an increase in SFA content from 16.34% to 17.77% was observed when incorporating larger amounts of ODS into the oleogel mixture. This could be attributed to the high content of SFA content present in ODS. A similar observation was reported by da Silva, et al. [11], who noted an increase in SFA content when palm stearin is added to olive oil. Shafika Abdul Kadir, et al. [23] revealed that the SFA content of diacylglycerol stearin–based biscuits is 22.72%, which surpasses that of OCW/ODS–based cookies. This result might be attributed to the inclusion of CW in the cookie formulation. The PUFA/SFA ratio is a nutritional index commonly employed to evaluate the impact of dietary intake on cardiovascular health (CVH). A higher value of this ratio indicates a more favorable effect [24]. As shown in Table 1, the PUFA/SFA ratios of OCW/ODS–based cookies were all higher than those of shortening cookies. These results indicated that OCW/ODS–based cookies might demonstrate a favorable impact on cardiovascular health.

### 3.2. Microstructure of Cookies

**Figure 1:**
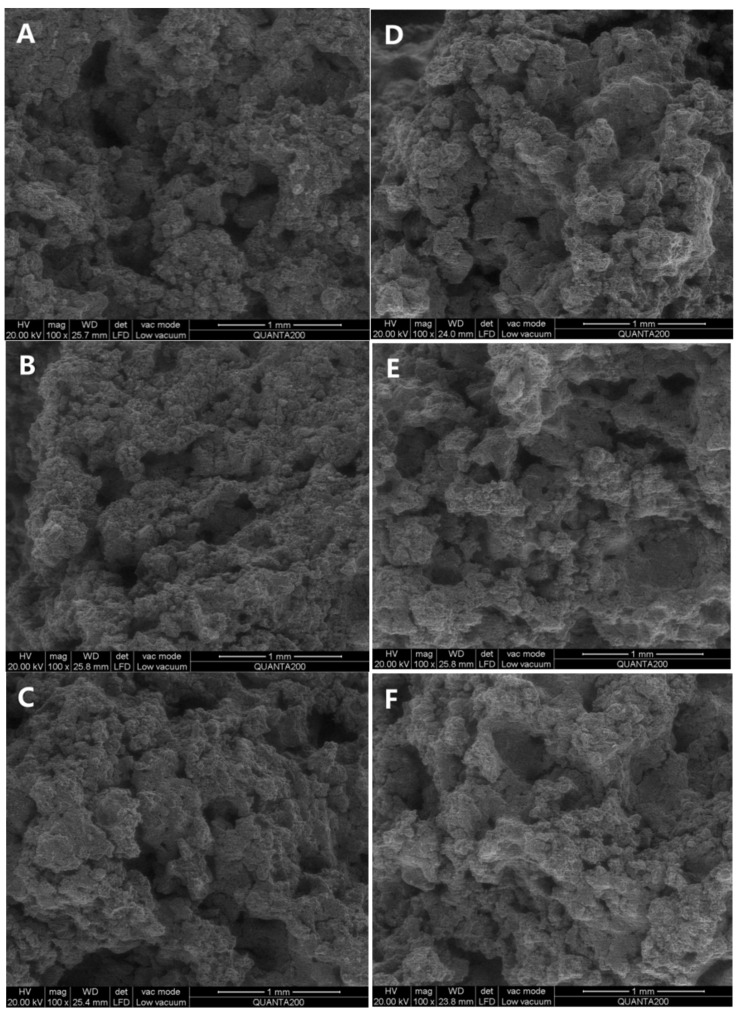
Microscopic morphology of shortening and OCW/ODS–based cookies. (**A**) shortening cookie, (**B**) OCW cookie, (**C**) OCW/ODS–5 cookie, (**D**) OCW/ODS–15 cookie, (**E**) OCW/ODS–25 cookie, (**F**) OCW/ODS–35 cookie. OCW: olive diacylglycerol oil/candelilla wax. ODS: olive diacylglycerol stearin.

The cross–sectional morphology of cookies was examined using scanning electron microscopy (SEM) to investigate the formation of lipid–amylose complexes, as depicted in Figure 1. Compared to shortening cookies, the surface structure of OCW/ODS–25 and OCW/ODS–35 cookies changed from porous to smooth, ultimately resulting in a denser internal structure. These results might be attributed to the higher concentration of long–chain saturated fatty acids in OCW/ODS–25 and OCW/ODS–35 cookies, which resulted in the formation of lipid–amylose complexes. Previous studies have demonstrated that the microstructure of lipid–amylose complexes typically exhibits an irregular porous mesh structure [10,25]. Zhang, et al. [26] also revealed that the surface of lipid–amylose complexes exhibited a reduction in their porous structure as the concentration of long–chain saturated fatty acids (stearic acids) increased. The enhanced compactness observed in these cookies may contribute to their increased resistance against amylase digestion. Consequently, incorporating an appropriate content (25%) of ODS into OCW/ODS–based cookies may potentially reduce the digestibility of cookie starch.

### 3.3. Oxidative Stability of Cookies

**Figure 2:**
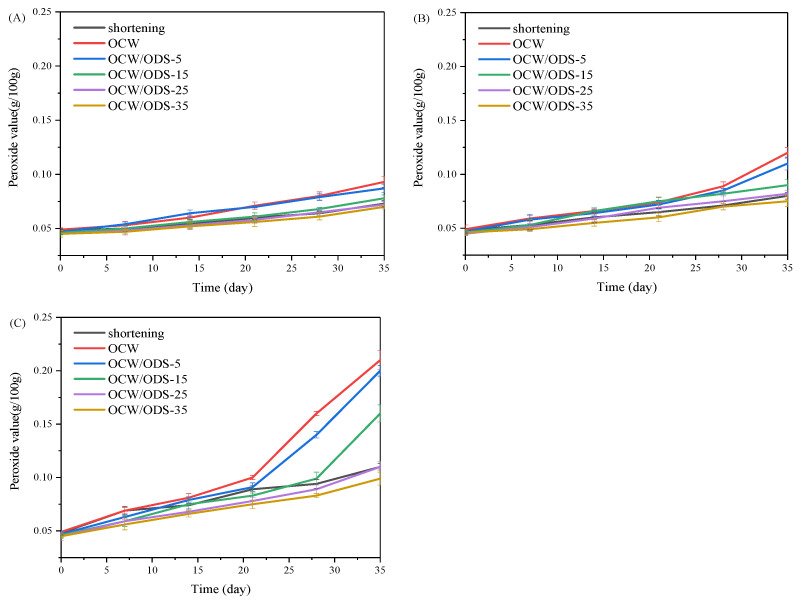
Peroxide value of shortening and OCW/ODS–based cookies at (**A**) 25 °C, (**B**) 35 °C and (**C**) 45 °C. OCW: olive diacylglycerol oil/candelilla wax. ODS: olive diacylglycerol stearin.

The oxidation of oil is a crucial factor that contributes to the deterioration of cookies during storage [27]. According to GB 7100–2015, the permissible limit for the peroxide value in cookies is set at 0.25 g/100 g [18]. Therefore, the PV of cookies with shortening and different contents of OCW/ODS–based oleogels were investigated during storage at 25 °C, 35 °C and 45 °C. As depicted in Figure 2, the peroxide value of the OCW cookie was found to be the highest among all cookies. When stored at the same temperature and duration, both OCW and OCW/ODS–5 cookies showed a significant increase in peroxide value. However, a lower final peroxide value was observed in OCW/ODS–35 cookies (0.10 g/100 g) compared to that of shortening cookies (0.11 g/100 g). The results indicated that the incorporation of ODS into OCW/ODS–based oleogel in cookie formulation enhanced oxidative stability, potentially due to the increased addition of ODS resulting in the formation of amylose–lipid complexes in cookies. These findings were consistent with those of Millao, et al. [28], who discovered that the compact networks significantly hinder the penetration and diffusion of oxygen within the samples. Thus, the enhanced oxidative stability might be attributed to the formation of amylose–lipid complexes caused by the amylose in starch and the long–chain saturated fatty acids in OCW/ODS–based oleogel, resulting in a compact network within the cookie.

### 3.4. The Digestibility of Cookies

The cookies contain three types of starch: rapidly digestible starch (RDS), slowly digestible starch (SDS) and resistant starch (RS) [10,29]. Consumption of SDS significantly contributes to the maintenance of postprandial glycemic levels over an extended duration [30], thereby reducing the risk of type II diabetes. Therefore, it is considered favorable to increase the concentration of RS and SDS as an approach to regulating the levels of blood glucose and promoting gastrointestinal well–being in individuals [31].

The in vitro digestibility of the cookies was evaluated, and the content of different starch types is summarized in Table 2. The results presented in Table 2 demonstrated that the shortening cookies contained proportions of RDS, SDS and RS at levels of 28.70%, 22.70% and 48.53%, respectively. Among all cookies, the OCW/ODS–based cookies containing 5% ODS exhibited the highest RDS content at 34.97%, while displaying the lowest SDS content at 21.57%. However, the ODS content in OCW/ODS–based cookies increased from 0% to 25% and 35%. There was a significant decrease in RDS content to 12.16% and 14.90%, respectively, along with an increase in RS content of 3.73% and 9.30%. Notably, compared to shortening cookies, the OCW/ODS–25 and OCW/ODS–35 cookies resulted in a significantly reduced RDS digestion (*p* < 0.05). This occurrence can be ascribed to the elevated levels of long–chain saturated fatty acids in ODS, leading to the formation of more compact amylose–lipid complexes. As a result, these complexes enhanced resistance against amylase digestion. These results aligned with a report conducted by Liu, et al. [14], indicating that the lipid–amylose complexes led to a diminished vulnerability towards enzymatic degradation, consequently leading to a decline in the digestibility of starch.

**Figure 3:**
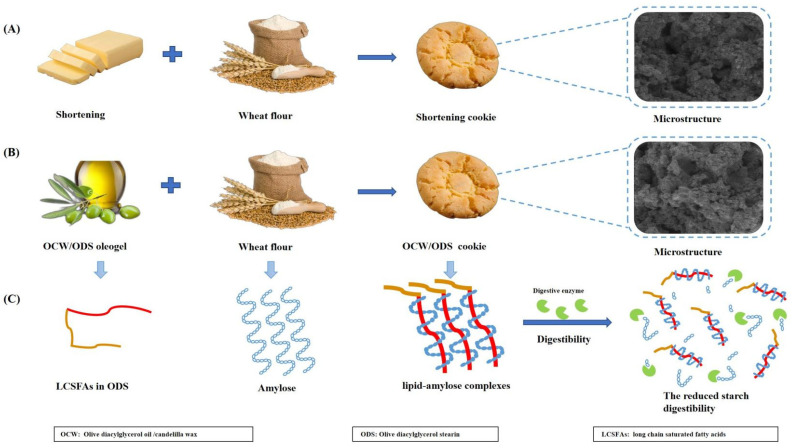
(**A**) The formulation and the microstructure of shortening cookie, (**B**) the formulation and the microstructure of OCW/ODS cookie, (**C**) the mechanism of the formation of lipid–amylose complexes and their ability to resist amylase digestion in OCW/ODS cookie.

However, our findings contrast with the study conducted by Barragán–Martínez, et al. [8], which found that replacing commercial shortening with canola oil/CW–based oleogel led to a significant increase in rapidly digestible starch (RDS) content by 56.67%, but also resulted in significant decreases of 14.01% and 71.58% in slowly digestible starch (SDS) and resistant starch (RS), respectively. Alvarez–Ramirez, et al. [20] also investigated the same results, revealing a significant increase in RDS content upon complete substitution of shortening with canola oil/CW oleogel, while SDS content remains unaltered. Our findings can be ascribed to the incorporation of ODS, which led to the formation of lipid–amylose complexes, subsequently mitigating the impact of CW–based oleogel on starch digestibility. The results reported by Chen, et al. [13] corroborated these findings, demonstrating that peanut diacylglycerol oil has the ability to form amylose–lipid complexes with OSA–starch. Figure 3 depicts the mechanism of the formation of lipid–amylose complexes and their ability to resist amylase digestion. To further confirm the existence of these complexes, we conducted comprehensive analyses including XRD, DSC and FTIR.

### 3.5. The Fundamental Mechanism of Oleogel on the Cookies Digestibility

#### 3.5.1. Crystalline Structure of Cookies

**Figure 4:**
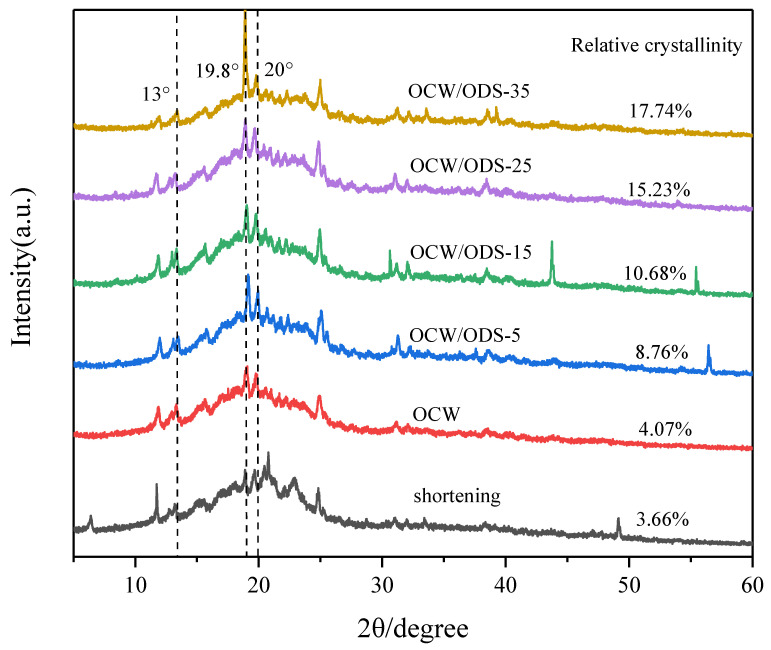
XRD patterns of shortening and OCW/ODS–based cookies. OCW: olive diacylglycerol oil/candelilla wax. ODS: olive diacylglycerol stearin.

XRD can be used to analyze the crystallization of cookies and understand the interaction between amylose in starch and lipid in OCW/ODS–based oleogels [32]. The formation of lipid–amylose complexes was indicated by the presence of diffraction peaks at around 13.3°, 19.8° and 20° in all cookies, as shown in Figure 4. These findings align with previous research conducted by Ahmed, et al. [32], which revealed the existence of complexes formed between lipids and amylose. These complexes exhibited diffraction peaks around 13.3°, 19.8° and 20°. The relative crystallinity is illustrated in Figure 4. Among the cookies, the shortening cookies exhibited the lowest relative crystallinity (3.66%). With an increase in ODS content, there was a gradual rise observed in the relative crystallinity. When the ODS content reached 35%, the relative crystallinity of lipid–amylose complexes was found to be 17.74%, representing a significant increase of 14.08% in comparison to the levels observed in shortening cookies. The findings suggested that the incorporation of ODS can enhance the formation of lipid–amylose complexes. As previously observed by Chen, et al. [13], peanut diacylglycerol stearin has the ability to form amylose–lipid complexes with OSA–starch. Zheng, et al. [33] also confirmed a strong propensity of diglycerides to form these complexes. Additionally, Zheng, et al. [33] have shown that an elevation in crystallinity leads to a decline in the rate of water diffusion and enzyme susceptibility, resulting in a decrease in an enhancement in SDS and RS content. Therefore, the higher ODS content may contribute to the reduced digestibility of starch in cookies.

#### 3.5.2. Gelatinization Properties of Cookies

The DSC analysis was utilized to examine the gelatinization characteristics of starch [34]. Parameters such as the enthalpy change (∆H_m_), final temperature (T_c_), peak temperature (T_p_) and initial temperature (T_o_) were employed to assess the starch gelatinization characteristics in cookies. Table 3 presents the behavior of starch gelatinization in shortening–based cookies and OCW/ODS–based cookies. The shortening cookie showed a gelatinization temperature range of 73.20 to 85.50 °C, with the highest point at 80 °C. These results aligned with our previous report, which demonstrated that the peak temperatures observed in peanut diacylglycerol oleogel–based cakes were at 68.4–70.6 °C [13]. When the ODS content reached 25% and 35%, the starch gelatinization of OCW/ODS–based cookies occurred over a wider temperature range, ranging from 69.70 °C to 85.30 °C and from 52.60 °C to 92.10 °C, respectively. The T_p_ was comparable to that of shortening cookies, while the ∆H_m_ exceeded that of shortening, reaching values of 67.78 J/g and 437.70 J/g, respectively. This observation can be ascribed to the fact that the complexes exhibited a higher degree of crystallinity, resulting in an increased ∆H_m_ [35], which was supported by the XRD analysis. This finding was consistent with the report by Henning, et al. [36], which demonstrated that starch exhibiting a higher concentration of complexes showed a greater ∆H_m_ during gelatinization.

#### 3.5.3. FTIR Spectra of Cookies

**Figure 5:**
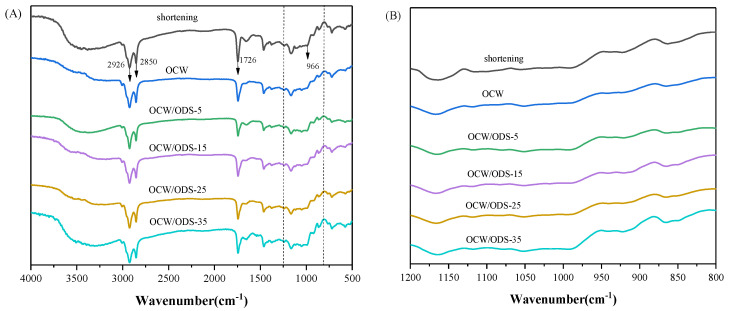
(**A**) Infrared spectra of shortening and OCW/ODS–based cookies and (**B**) the region (1200 to 800 cm^−1^) of shortening and OCW/ODS–based cookies. OCW: olive diacylglycerol oil/candelilla wax. ODS: olive diacylglycerol stearin.

FTIR spectroscopy was employed to examine the molecular interactions between amylose present in starch and lipids within OCW/ODS–based oleogels. The FTIR spectra of cookies are illustrated in Figure 5A. The significant peak detected within the range of 3200 cm^−1^ to 3500 cm^−1^ corresponds to the bending vibrations of N–H groups in gluten molecules [35]. The vibrations of C–H bonds were attributed to the significant peaks detected at 2850 cm^−1^ and 2926 cm^−1^ [31]. The vibration of the C=O bond was identified as the cause for the absorption peak detected at 1726 cm^−1^ [37]. The absorption peak observed at 966 cm^−1^ was attributed to the deformation band of *trans* fatty acids [38]. The FTIR spectrum of the shortening cookie revealed a specific peak at 966 cm^−1^, indicating the presence of *trans* fatty acids in the shortening cookie. Figure 5B depicts the region (1200 cm^−1^ to 800 cm^−1^) of starch molecules, with 1047 cm^−1^ characterizing the crystalline domain, 1022 cm^−1^ characterizing the amorphous domain and 995 cm^−1^ characterizing hydrogen bond strength between starch molecules. The ratio of peak intensities at 1047 cm^−1^ and 1022 cm^−1^ (R_1047/1022_) serves as a measure for characterizing the degree of the short–range order in starch. A higher R_1047/1022_ value indicates a more pronounced short–range ordered structure [39] and enhanced resistance to amylase digestion [40,41]. The intensity ratio between peaks at 1022 cm^−1^ and 995 cm^−1^ (R_1022/995_) provides insights into the strength of hydrogen bonding within starch molecules, where a decreased intensity ratio indicates enhanced intermolecular hydrogen bond forces [42].

**Figure 6:**
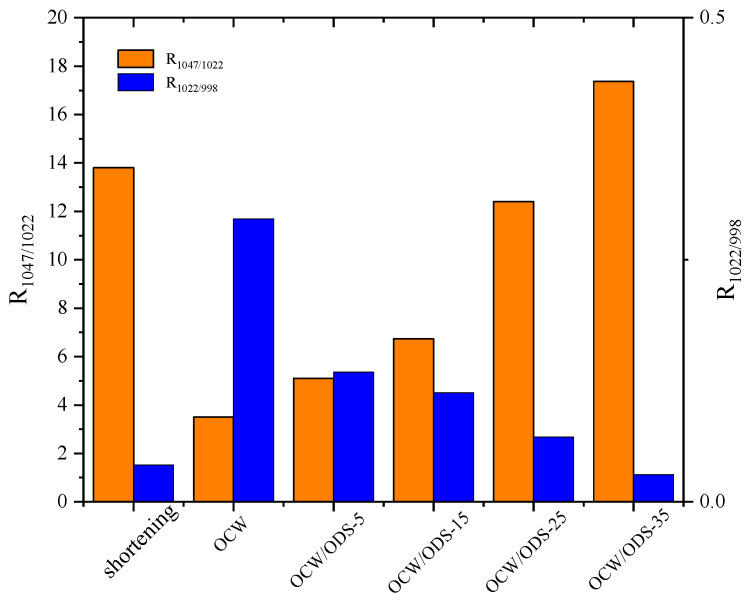
Peak intensity ratio of shortening and OCW/ODS–based cookies. R_1047/1022_ represents the intensity ratio of the bands at 1047 and 1022 cm^−1^, R_1022/998_ represents the intensity ratio of the bands at 1022 and 998 cm^−1^. OCW: olive diacylglycerol oil/candelilla wax. ODS: olive diacylglycerol stearin.

The R_1047/1022_ value, as shown in Figure 6, was observed to be highest in the OCW/ODS–35 cookies (17.37), exhibiting a significant increase of 20.55% compared to that of shortening cookies. Conversely, the OCW cookie without ODS displayed the lowest intensity at 3.50. Moreover, there was a gradual increase in the peak intensity within OCW/ODS–based cookies as the ODS content increased. These results indicated that the incorporation of different ODS contents in OCW/ODS–based oleogel could enhance the formation of lipid–amylose complexes. These results align with a study by Wang, et al. [39], which showed that the increased crystallinity of lipid–amylose complexes has the potential to enhance the short–range structural ordering of starch. However, the observed trend in the R_1047/1022_ and R_1022/998_ values exhibited diametrically opposite behavior, indicating that an increased ODS content enhanced hydrogen bonding forces in OCW/ODS–based cookies. This enhancement can be attributed to the hindered interaction between starch and CW, which was caused by the emulsibility of ODS. The present observation aligned with the findings of a previous study conducted by Muscat, et al. [43], which demonstrated that the presence of an emulsifier hinders the interaction between starch and beeswax, leading to an increased occurrence of hydrogen bonding reactions among starch molecules. Overall, the hydrogen bonding forces of the OCW/ODS–35 cookies were found to be relatively stronger, accompanied by a structure that displayed short–range ordering. Previous studies by Wang, et al. [39] suggested that these stronger forces and ordered structures may result in reduced starch digestibility.

## 4. Conclusions

The present study revealed that the inclusion of OCW/ODS–based cookies resulted in reduced starch digestibility and enhanced oxidative stability compared to cookies containing shortening. The in vitro digestibility revealed that the OCW/ODS–based cookies exhibited a significant 17.74% enhancement in SDS and RS contents when compared with shortening cookies, which can be attributed to the increased degree of crystallinity caused by lipid–amylose complexes. OCW/ODS–based cookies exhibited a greater change in gelatinization enthalpy, potentially attributed to the complexes still maintaining a higher level of crystallinity. FTIR analysis showed that the incorporation of ODS facilitated the lipid–amylose complexes formation, possibly owing to the presence of long–chain saturated fatty acids in ODS. Hence, these findings demonstrated that utilizing OCW/ODS–based oleogels was a viable alternative for replacing shortenings in cookie formulations.

## Figures and Tables

**Table 1:** The fatty acid composition and the fatty acid total amount of shortening, ODO, ODS and OCW/ODS–based oleogels. SFA represents saturated fatty acid, MUFA represents monounsaturated fatty acid and PUFA represents polyunsaturated fatty acid. TFA represents *trans* fatty acid. ODO: olive diacylglycerol oil, ODS: olive diacylglycerol stearin, OCW: olive diacylglycerol oil/candelilla wax.

Sample		Shortening	ODO	ODS	OCW	OCW/ODS–15	OCW/ODS–25	OCW/ODS–35
Fatty acid composition (%)	C6–15:0	27.23 ± 0.01 ^a^	–	–	–	–	–	–
	C16:0	32.48 ± 0.01 ^a^	12.82 ± 0.03 ^g^	20.24 ± 0.03 ^b^	13.36 ± 0.02 ^f^	13.90 ± 0.03 ^e^	14.26 ± 0.04 ^d^	14.52 ± 0.06 ^c^
	C16:1	2.16 ± 0.01 ^a^	0.91 ± 0.09 ^b^	0.83 ± 0.05 ^c^	0.91 ± 0.02 ^b^	0.91 ± 0.01 ^b^	0.9 ± 0.01 ^b^	0.91 ± 0.01 ^b^
	C18:0	10.12 ± 0.02 ^a^	3.08 ± 0.01 ^g^	5.17 ± 0.01 ^b^	3.23 ± 0.02 ^f^	3.39 ± 0.01 ^e^	3.47 ± 0.03 ^d^	3.55 ± 0.02 ^c^
	C18:1n9t	3.73 ± 0.01 ^a^	–	–	–	–	–	–
	C18:1n9c	20.65 ± 0.02 ^g^	69.69 ± 0.01 ^a^	63.26 ± 0.01 ^f^	69.29 ± 0.00 ^b^	68.72 ± 0.05 ^c^	68.37 ± 0.01 ^d^	68.2 ± 0.01 ^e^
	C18:2n6c	1.11 ± 0.01 ^g^	11.8 ± 0.01 ^a^	8.83 ± 0.12 ^f^	11.5 ± 0.03 ^b^	11.42 ± 0.02 ^c^	11.24 ± 0.02 ^d^	11.09 ± 0.01 ^e^
	C20:0	–	0.44 ± 0.02 ^d^	0.65 ± 0.02 ^a^	0.46 ± 0.01 ^c^	0.48 ± 0.00 ^b^	0.48 ± 0.00 ^b^	0.49 ± 0.01 ^b^
	C20:1	1.33 ± 0.01 ^a^	1.27 ± 0.02 ^b^	0.96 ± 0.01 ^e^	1.24 ± 0.02 ^c^	1.24 ± 0.01 ^c^	1.22 ± 0.01 ^cd^	1.21 ± 0.00 ^d^
Fatty acid total amount (%)	SFA	69.83 ± 0.03 ^a^	16.34 ± 0.02 ^g^	26.06 ± 0.01 ^b^	17.05 ± 0.04 ^f^	17.77 ± 0.02 ^e^	18.21 ± 0.02 ^d^	18.21 ± 0.03 ^c^
	MUFA	27.87 ± 0.02 ^g^	71.87 ± 0.11 ^a^	65.05 ± 0.04 ^f^	71.44 ± 0.03 ^b^	70.87 ± 0.04 ^c^	70.5 ± 0.01 ^d^	70.32 ± 0.01 ^e^
	PUFA	1.11 ± 0.01 ^g^	11.8 ± 0.01 ^a^	8.83 ± 0.10 ^f^	11.5 ± 0.03 ^b^	11.42 ± 0.02 ^c^	11.24 ± 0.02 ^d^	11.09 ± 0.01 ^e^
Nutritional indexes	PUFA/SFA	1.59 ± 0.02	72.20 ± 0.01	33.89 ± 0.45	67.47 ± 0.31	64.25 ± 0.06	61.73 ± 0.14	59.78 ± 0.25
	TFA	3.73 ± 0.01	–	–	–	–	–	–

Data are expressed as mean ± standard deviation (n = 3) and different letters in the same column show significant difference at the 5% level in Duncan’s test (*p* < 0.05).

**Table 2:** Rapidly digestible starch (RDS), slowly digestible starch (SDS) and resistant starch (RS) of shortening and OCW/ODS–based cookies. OCW: olive diacylglycerol oil/candelilla wax. ODS: olive diacylglycerol stearin.

Sample	RDS	SDS	RS
shortening	28.70 ± 1.01 ^d^	22.70 ± 0.95 ^d^	48.53 ± 0.71 ^b^
OCW	31.63 ± 0.91 ^b^	25.53 ± 0.74 ^c^	42.37 ± 0.85 ^de^
OCW/ODS–5	34.97 ± 0.72 ^a^	21.57 ± 0.95 ^d^	43.50 ± 0.90 ^d^
OCW/ODS–15	32.90 ± 0.75 ^a^	24.57 ± 0.75 ^c^	42.57 ± 0.86 ^de^
OCW/ODS–25	19.47 ± 0.86 ^e^	28.77 ± 0.46 ^b^	46.10 ± 0.70 ^c^
OCW/ODS–35	16.73 ± 0.96 ^f^	37.30 ± 0.91 ^a^	51.67 ± 0.85 ^a^

Data are expressed as mean ± standard deviation (n = 3) and different letters show significant differences at the 5% level in Duncan’s test (*p* < 0.05).

**Table 3:** The initial temperature (T_o_), peak temperature (T_p_), final temperature (T_c_), enthalpy change (∆H_m_) of gelatinization of shortening and OCW/ODS–based cookies. OCW: olive diacylglycerol oil/candelilla wax. ODS: olive diacylglycerol stearin.

Sample	T_o_ (°C)	T_p_ (°C)	T_c_ (°C)	∆H_m_ (J/g)
shortening	73.20	80.00	85.50	2.77
OCW	–	–	–	–
OCW/ODS–5	81.90	83.30	86.20	0.90
OCW/ODS–15	86.40	91.50	93.90	1.17
OCW/ODS–25	69.70	76.60	85.30	67.78
OCW/ODS–35	52.60	80.00	92.10	437.70

## Data Availability

The original contributions presented in the study are included in the article, further inquiries can be directed to the corresponding author.
